# Targeting heparanase overcomes chemoresistance and diminishes relapse in myeloma

**DOI:** 10.18632/oncotarget.6408

**Published:** 2015-11-27

**Authors:** Vishnu C. Ramani, Fenghuang Zhan, Jianbo He, Paola Barbieri, Alessandro Noseda, Guido Tricot, Ralph D. Sanderson

**Affiliations:** ^1^ Department of Pathology, University of Alabama at Birmingham, Birmingham, AL, USA; ^2^ Comprehensive Cancer Center, University of Alabama at Birmingham, Birmingham, AL, USA; ^3^ Department of Internal Medicine, University of Iowa, Iowa City, IA, USA; ^4^ Holden Comprehensive Cancer Center, University of Iowa, Iowa City, IA, USA; ^5^ Sigma-tau Research Switzerland S.A., Mendrisio, Switzerland

**Keywords:** heparanase, multiple myeloma, roneparstat, drug resistance, chemotherapy

## Abstract

In most myeloma patients, even after several rounds of intensive therapy, drug resistant tumor cells survive and proliferate aggressively leading to relapse. In the present study, gene expression profiling of tumor cells isolated from myeloma patients after sequential rounds of chemotherapy, revealed for the first time that heparanase, a potent promoter of myeloma growth and progression, was elevated in myeloma cells that survived therapy. Based on this clinical data, we hypothesized that heparanase was involved in myeloma resistance to drug therapy. In several survival and viability assays, elevated heparanase expression promoted resistance of myeloma tumor cells to chemotherapy. Mechanistically, this enhanced survival was due to heparanase-mediated ERK signaling. Importantly, use of the heparanase inhibitor Roneparstat in combination with chemotherapy clearly diminished the growth of disseminated myeloma tumors *in vivo*. Moreover, use of Roneparstat either during or after chemotherapy diminished regrowth of myeloma tumors *in vivo* following therapy. These results provide compelling evidence that heparanase is a promising, novel target for overcoming myeloma resistance to therapy and that targeting heparanase has the potential to prevent relapse in myeloma and possibly other cancers.

## INTRODUCTION

Multiple myeloma is a B cell malignancy characterized by destructive bone lesions, chemoresistance, tumor relapse and poor patient outcome [1]. Because myeloma is incurable and the disease relapses in almost all patients, different therapeutic strategies are being explored to target relapse and improve patient outcome [2, 3]. Evidence indicates that the myeloma tumor microenvironment plays an important role in driving resistance that leads to relapse [4, 5]. Heparanase, by elevating the expression and activity of growth factors (HGF, VEGF), proteases (MMP-9, uPA) and RANKL, primes the tumor microenvironment to favor myeloma growth and dissemination [6–8]. This is consistent with the well-established roles for heparanase in driving inflammation and the progression of different tumor types [9]. Heparanase is present in the bone marrow of most myeloma patients where high levels of heparanase enzyme activity correlates with elevated angiogenic activity, an important promoter of myeloma growth and progression [10]. Targeting heparanase activity using Roneparstat, a modified heparin that is 100% N-acetylated and 25% glycol split, clearly diminishes aggressive myeloma growth *in vivo* [11]. This efficacy of Roneparstat is due, at least in part, to down regulation of HGF, VEGF and MMP-9 expression *in vivo*, all of which are known to be driven by heparanase [12]. Despite this clear evidence that heparanase is an important driver of myeloma progression, a role for heparanase in myeloma drug resistance has not been addressed.

The present work demonstrates that tumor cells that resist and survive therapy in myeloma patients have elevated levels of heparanase. Probing a role for heparanase in myeloma resistance we found that heparanase, by maintaining high levels of active ERK in tumor cells, promotes tumor survival that drives resistance in response to therapy. In cell-based models and in animal models of disseminated myeloma, use of the heparanase inhibitor Roneparstat both in combination with or after chemotherapy clearly diminished tumor burden and improved the overall outcome of therapy. These studies collectively provide novel evidence for heparanase directing the outcome of chemotherapy in favor of tumor relapse and strongly validate the incorporation of heparanase inhibition as a therapeutic strategy against myeloma.

## RESULTS

### Tumor cells that survive intensive chemotherapy in myeloma patients express high heparanase

**Figure 1:**
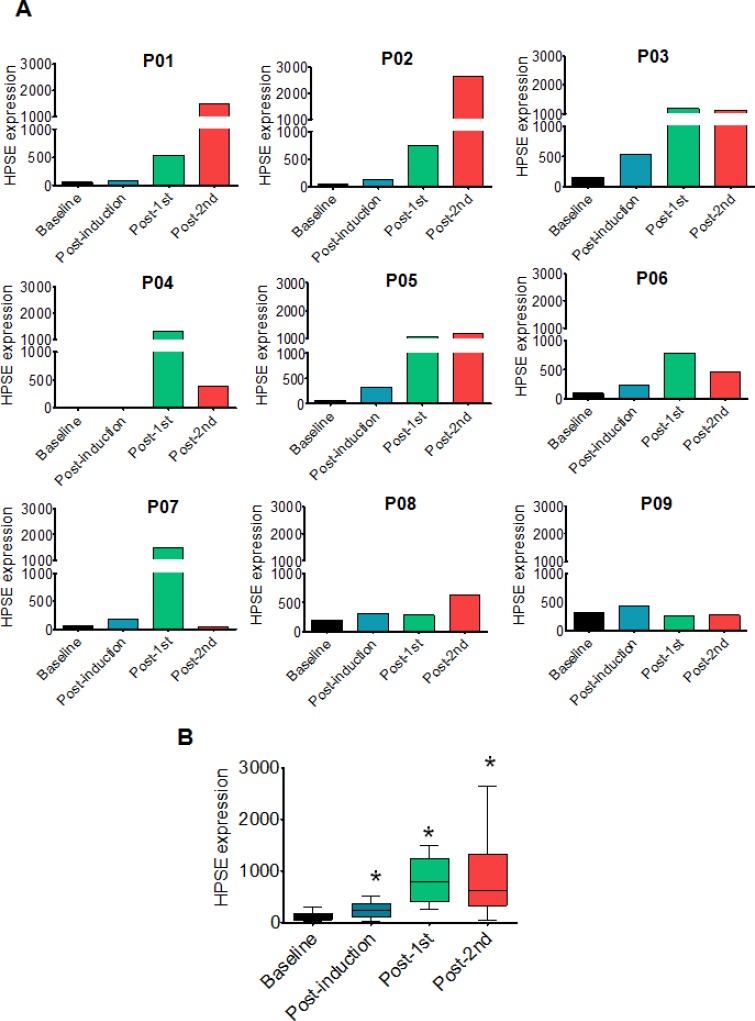
Heparanase expression is high in primary myeloma tumor cells that survive and grow following chemotherapy (**A**) Heparanase expression in purified tumor cells from nine individual myeloma patients (designated P01-P09) at specific times during the course of therapy (baseline, post-induction, post-1st transplant, post-2nd transplant) as determined by gene expression profiling (please refer to Materials and Methods for details). (**B**) Average expression of heparanase from nine myeloma patients after sequential rounds of chemotherapy. *p < 0.05 versus baseline.

Gene expression profiling of tumor cells from myeloma patients revealed that heparanase expression was high in the cells that survived and grew following chemotherapy (Figure 1A). In all the patients tested, heparanase expression was low prior to therapy (baseline) but was clearly elevated in most patients after chemotherapy (Figure 1A). Compared to baseline, the average heparanase expression in primary myeloma tumors was significantly elevated over consecutive rounds of therapy (Figure 1B). In 7/9 patients, compared to their corresponding baseline levels, the fold increase in heparanase expression was markedly higher after the second round of chemotherapy (Supplementary Table 1).

### Heparanase enhances myeloma drug resistance

**Figure 2:**
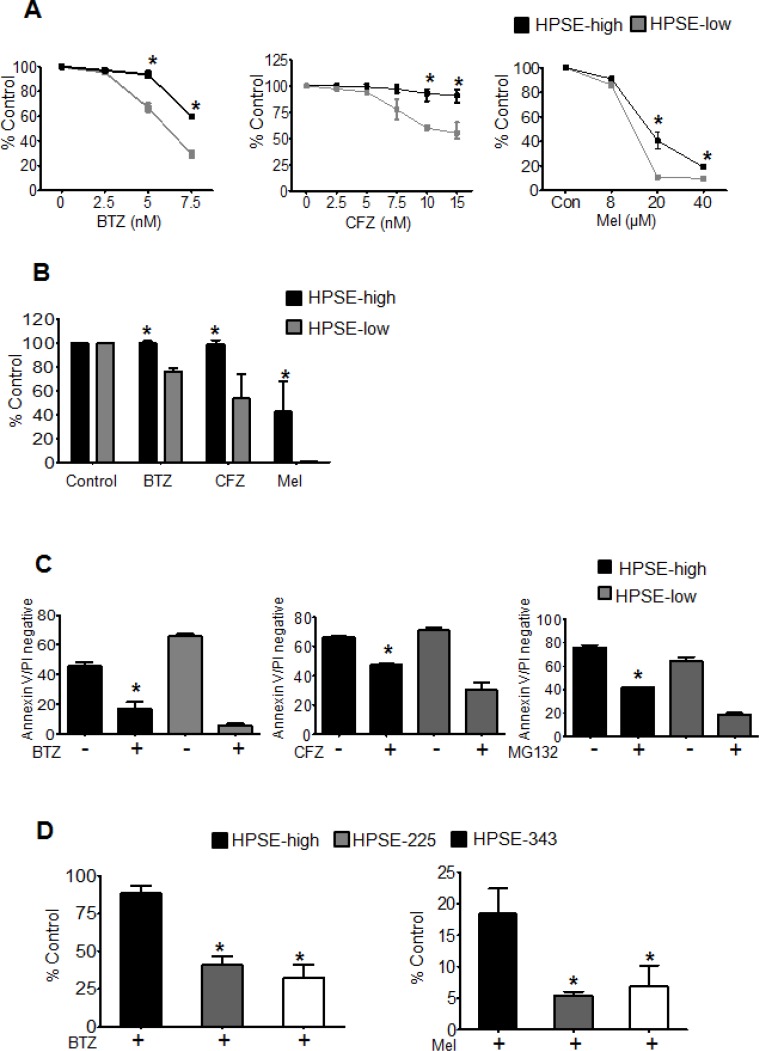
Heparanase promotes chemoresistance (**A**) Stable transfectants of CAG cells expressing either high (HPSE-high) or low (HPSE-low) levels of heparanase were treated with increasing concentrations of bortezomib (BTZ), carfilzomib (CFZ) or melphalan (Mel) for 14 h and cell viability was assessed by MTT assay. *p < 0.05 versus HPSE-low. (**B**) Differences in cell viability between HPSE-high and HPSE-low after 14 h, treatment with BTZ (5 nM), CFZ (7.5 nM), or Mel (40 μM) as determined by ATPlite^™^ assay, *p < 0.05 versus HPSE-low. (**C**) Equal numbers (10^6^ cells/ml) of HPSE-high or HPSE-low cells were treated for 14 h with BTZ (50 nM), CFZ (100 nM) or another proteasome inhibitor MG132 (100 nM) and the percentage of viable cells (Annexin V and Propidium Iodide negative) was determined by flow cytometry, *p < 0.05 versus HPSE-low after drug treatment. (**D**) Viability of CAG HPSE-high cells and CAG cells expressing enzymatically inactive HPSE (mutations at amino acids 225 or 343; HPSE-225, HPSE-343) as measured by MTT assay after 14 h treatment with BTZ (5 nM) or Mel (40 uM), *p < 0.05 versus HPSE-high. Data are represented as mean ± SEM.

Because heparanase was associated with the tumor cells that survive chemotherapy, we speculated that it was involved in myeloma resistance to therapy. To test this we treated cells having different levels of heparanase expression with different anti-myeloma drugs, bortezomib (BTZ), carfilzomib (CFZ) or melphalan (Mel) for 14 h and assessed their viability by MTT assay and ATPlite^™^ viability assay. HPSE-high and HPSE-low CAG human myeloma cells exhibit a 4-fold difference in their levels of heparanase and have levels comparable to those found in the bone marrow of many myeloma patients [10]. The HPSE-high cells have been characterized extensively in these previous studies and they represent a physiologically relevant model for studying heparanase function in myeloma. In both the cell viability assays and against different doses of therapeutic agents, HPSE-high cells demonstrated significantly higher cell viability compared to HPSE-low cells (Figure 2A, 2B). Staining for Annexin V (a marker of apoptosis), confirmed the cells surviving after 14 h drug treatment are truly a viable population (Annexin V and PI negative) and not cells in early stages of apoptosis (Figure 2C). To determine if heparanase enzyme activity was required for heparanase- enhanced drug resistance, we compared the viability of CAG cells expressing mutated, enzymatically inactive forms of heparanase (HPSE-225, HPSE-343) to HPSE-high cells. HPSE-225 and HPSE-343 express the mutant heparanase enzyme at levels comparable to the heparanase expressed in HPSE-high cells [13]. To determine if heparanase enzymatic activity confers resistance against different classes of chemotherapeutic drugs, we examined cell response to treatment with bortezomib (proteasome inhibitor) or melphalan (alkylating agent). After 14 h treatment with bortezomib or melphalan, HPSE-high cells had significantly higher viability than the cells expressing mutated heparanase thereby demonstrating the importance of heparanase enzymatic activity in myeloma cell resistance to chemotherapy (Figure 2D).

### Blocking heparanase-driven ERK signaling sensitizes myeloma cells to chemotherapy

**Figure 3:**
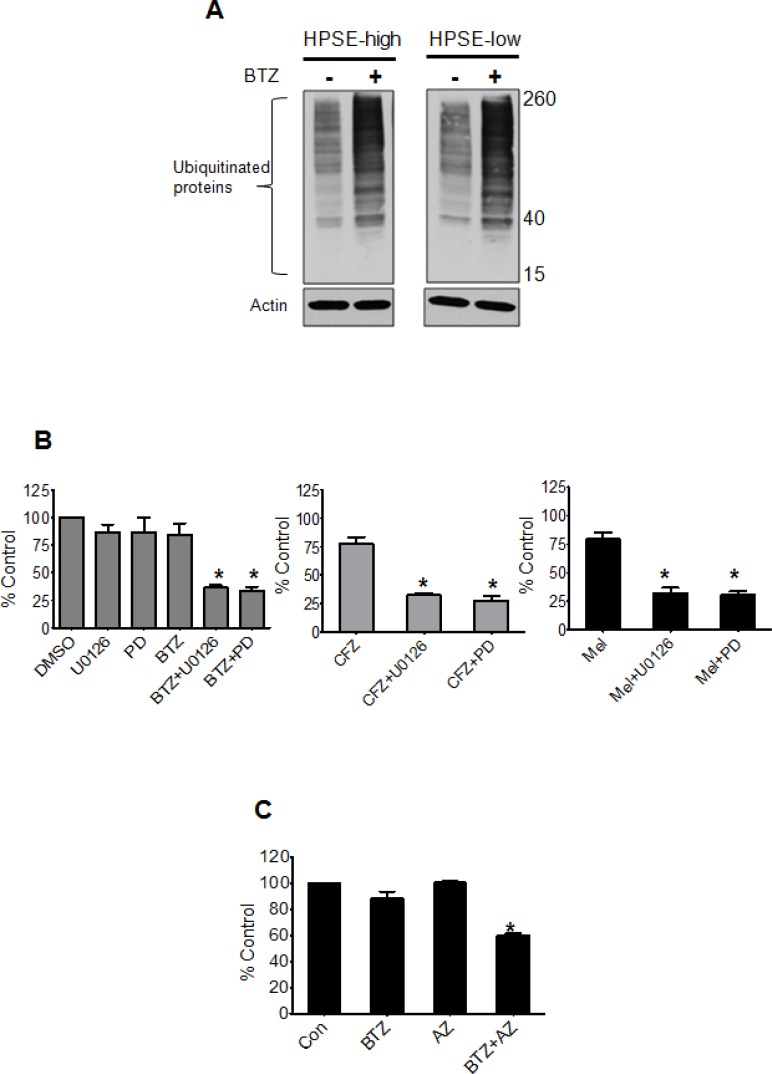
Blocking ERK overcomes heparanase-induced chemoresistance of myeloma cells (**A**) Western blots of ubiquitinated protein in cell extracts from HPSE-high and HPSE-low after overnight incubation with BTZ (10 nM). Actin served as the loading control. (**B**) Viability of HPSE-high cells treated with ERK inhibitors U0126 (25 μM) or PD98059 (PD, 50 μM) for 2 h prior to incubation with BTZ (5 nM), CFZ (7.5 nM) or Mel (10 μM) for 14 h. PD98059 at a concentration of 50 uM is shown to block ERK mediated signaling in our previous studies involving HPSE-high cells [14]. Controls included cells treated with ERK inhibitors or chemotherapeutic drugs alone. Viability was determined by MTT assay, *p < 0.05 versus individual drug treatment alone. (**C**) Viability of HPSE-high cells treated with either BTZ (5 nM) or MEK inhibitor- AZD6244 (AZ) (200 nM) alone or a combination of the two drugs for 14 h, *p < 0.005 versus BTZ treatment alone. Data are represented as mean ± SEM.

To identify the molecular mechanism by which heparanase drives drug resistance, we first tested whether the target of drug therapy is altered by heparanase. Bortezomib targets the proteasome resulting in accumulation of ubiquitinated proteins in myeloma cells. Overnight treatment of HPSE-high and HPSE-low cells with bortezomib resulted in similar levels of accumulated ubiquinated proteins confirming that the level of heparanase did not affect the proteasome (Figure 3A). We previously demonstrated that HPSE-high cells have much higher levels of active extracellular signal-regulated kinase (ERK) compared to HPSE-low cells [14]. This is important because activation of ERK in response to different stimuli is implicated in myeloma tumor survival and drug resistance [15], making the ERK pathway a very attractive therapeutic target [16]. Consistent with a role for ERK in heparanase-driven resistance, blocking ERK activation using MEK inhibitors (U0126, PD98059, and Selumetinib (AZD 6244)), significantly decreased the resistance of HPSE-high cells to BTZ (5 nM), CFZ (7.5 nM), and Mel (10 μM) (Figure 3B, 3C).

### Heparanase inhibitor Roneparstat sensitizes tumors to chemotherapy and prevents relapse

**Figure 4:**
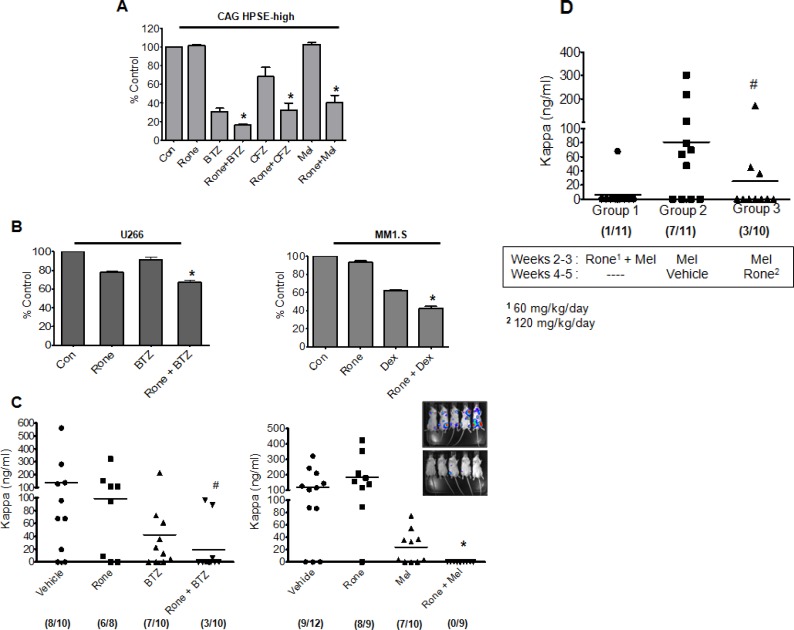
Roneparstat sensitizes myeloma cells to chemotherapy and diminishes relapse (**A**) MTT assay of HPSE-high cells treated with BTZ (7.5 nM), CFZ (15 nM), or Mel (8 μM) for 14 h either alone or following 6 h pretreatment with heparanase inhibitor, Roneparstat (Rone, 6.75 μM), *p < 0.05 versus individual drug treatment alone. (**B**) (Left panel) MTT assay for myeloma cell line U266 treated with BTZ (5 nM) for 14 h either alone or following 6 h pretreatment with Roneparstat (6.75 μM), *p < 0.05 versus BTZ alone. (Right panel) MTT assay for dexamethasone sensitive myeloma cell line MM1.S treated with dexamethasone (Dex, 50 nM) for 14 h either alone or following 8 h pretreatment with Roneparstat (6.75 μM), *p < 0.05 versus Dex alone. (**C**) Disseminated tumors were established in SCID mice by intravenous injection of CAG HPSE-high cells and after 7 days animals were sorted into different groups and treatment was initiated. After 14 days of treatment, tumor burden in individual animals was determined by quantification by ELISA of human kappa immunoglobulin light chain in murine sera. Different treatment groups included (Left panel) Roneparstat (120 mg/kg/day) alone, BTZ (0.5 mg/kg/twice weekly) alone, or a combination of Roneparstat (120 mg/kg/day) and BTZ (0.5 mg/kg/twice weekly). We analyzed the combination therapy group for potential outliers using the statistical analyses tool called ROUT (Graph Pad software), that identifies outliers from a nonlinear regression. The maximum false discovery rate (Q) for ROUT test was set to maximum at 0.1000%. In the combination group, the two animals with high kappa levels were found to be outliers by ROUT analyses. However, to avoid any biased interpretation as well as to provide all the available data to the readers, we have not removed these outliers from our results. Due to inclusion of these outliers in the Rone + BTZ group, this group is not significantly different than the BTZ treatment alone group. However, if these two outliers are removed, the combination group is significantly different (*p* = 0.0065) compared to BTZ alone. Also note that only 3/10 animals in the combination group had detectable tumor vs. 7/10 in the BTZ alone group, #*p* < 0.05 versus animals treated with Rone alone. (Right panel) Roneparstat (60 mg/kg/day) alone, Mel (1.0 mg/kg/week) alone, or a combination of Roneparstat (60 mg/kg/day) and Mel (1.0 mg/kg/week). Animals bearing tumors treated with vehicle alone served as controls, *p < 0.05 versus animals treated with Mel alone. Numbers enclosed in parenthesis below each group denotes the number of animals with detectable tumor/total number of animals in the group. Inset – Bioluminescent images of disseminated myeloma tumors growing in bone of animals belonging to the vehicle group (top) and combination group (bottom). Images were taken after 14 days of treatment. (**D**) Disseminated tumors were established in SCID mice by intravenous injection of HPSE-high cells and after 7 days, animals were sorted into different treatment groups. Tumor burden for all the groups at the end of experiment (5 weeks) was determined by quantification of human kappa immunoglobulin light chain in murine sera using ELISA. Melphalan concentration was 2.5 mg/kg/week in all groups; Roneparstat concentration was 60 mg/kg/day in Group 1 and 120 mg/kg/day in Group 3. #*p* < 0.05 versus Group 2. Numbers enclosed in parenthesis below each group denotes the number of animals with detectable tumor burden/total number of animals in the group.

Because our data indicate that heparanase promotes drug resistance of myeloma cells, we tested whether blocking heparanase enzyme activity using heparanase inhibitor, Roneparstat (Rone) would sensitize myeloma cells to chemotherapy. Myeloma cells from three different cell lines (CAG HPSE-high, U266 and MM1.S) were treated with different chemotherapeutic agents for 14 h either alone or following 6 h pretreatment with heparanase inhibitor, Roneparstat (Rone, 6.75 μM). Results revealed that Roneparstat in combination with these drugs significantly decreased cell viability compared to drug treatment alone (Figure 4A, 4B).

Next, we tested Roneparstat in combination with bortezomib or melphalan following intravenous injection of CAG HPSE-high cells. This is a highly aggressive myeloma model in which tumor cells disseminate and grow predominantly in bone, therefore closely mimicking aspects of the human disease (Inset, Figure 4C). Compared to animals treated with Roneparstat, bortezomib or melphalan alone, animals receiving the combination of Roneparstat and chemotherapy exhibited dramatically reduced tumor burden (Figure 4C). Notably, tumor burden was below the threshold of detection in many animals when Roneparstat was included in the combination (Figure 4C, numbers in brackets below each group indicate the number of animals with detectable tumor burden). Quantification of tumor growth by bioluminescent imaging further validated these findings (Supplementary Data, Figure 1). This suggests that blocking heparanase activity in the presence of anti-myeloma drugs dramatically decreases chemoresistance to diminish myeloma tumor survival. Because of the effectiveness of the melphalan/Roneparstat combination, we performed a final experiment in which animals bearing tumors were treated with the combination (melphalan/Rone) for two weeks then examined for tumor burden two weeks after treatment ended (Figure 4D, Group 1). Only one animal exhibited tumors raising the possibility that the melphalan/Roneparstat combination had eradicated tumors in some animals. In addition, we tested whether treatment with Roneparstat after melphalan therapy (rather than in combination with melphalan) would diminish tumor reoccurrence. For this experiment, tumors were treated for two weeks with melphalan, followed by two weeks of treatment with Roneparstat (Figure 4D Group 3) or vehicle (Figure 4D Group 2). Results demonstrated that Roneparstat significantly diminished reoccurrence of tumors compared to animals treated with vehicle control, indicating that Roneparstat was efficacious in diminishing relapse.

## DISCUSSION

The present study demonstrates that heparanase promotes drug resistance and relapse in myeloma. In support of these conclusions we found that; i) in myeloma patients, tumor cells that survive sequential rounds of chemotherapy express high levels of heparanase, ii) high heparanase promotes chemoresistance via ERK signaling, iii) the heparanase inhibitor Roneparstat sensitizes myeloma cells to chemotherapy, and iv) Roneparstat has the potential to prevent regrowth of tumors after chemotherapy. Together these studies reveal the importance of heparanase in promoting chemoresistance and provide rationale for the clinical use of the heparanase inhibitor Roneparstat in combination with other anti-myeloma drugs.

The discovery that heparanase expression is high in the cells that survive chemotherapy in myeloma patients (Figure 1) also supports the notion that heparanase plays an important role in drug resistance *in vivo*. It is possible that chemotherapy selectively kills those cells having low heparanase expression, leaving cells with high heparanase to subsequently grow and expand. This is supported by our finding that cells with high heparanase are more resistant *in vitro* to anti-myeloma drugs than are cells with low heparanase expression (Figure 2). However, it is also possible that chemotherapy itself stimulates heparanase expression which in turn enhances survival of the treated cells. In fact, we have found that anti-myeloma drugs do promote heparanase expression and secretion in myeloma cell lines (unpublished observation). Follow-up studies on this phenomenon are currently underway.

Our finding that heparanase drives ERK signaling to promote drug resistance (Figure 3) is consistent with the known role of ERK in regulating myeloma cell proliferation, survival, drug resistance, and angiogenesis [15, 17]. Importantly, activation of the ERK pathway is dependent on the enzyme activity of heparanase [14]. This explains why cells expressing an inactive form of the heparanase enzyme and therefore having low levels of active ERK exhibit diminished survival after drug treatment compared to HPSE-high cells (Figure 2D). Although little is known regarding the role of heparanase in drug resistance, it was recently reported that lapatinib-resistant breast cancer cell lines express high levels of heparanase and use of the heparanase inhibitor Roneparstat sensitized the drug resistant breast cancer cells to lapatinib [18]. Moreover, ERK inhibition was also observed when lapatinib resistant breast cancer cells were treated with Roneparstat [18], consistent with our previous finding that Roneparstat blocks heparanase-induced ERK signaling [12]. In addition to these studies on Roneparstat, another heparanase inhibitor, PG545, was recently shown to enhance the anticancer activity of chemotherapies in animal models of ovarian and pancreatic cancer [19, 20]. Together with our current findings, these studies support the notion that heparanase may contribute to the promotion of drug resistance in many types of cancer.

The surge in interest in heparanase inhibitors over the last decade reflects the recognition within the field that heparanase plays a vital role in cancer pathogenesis and progression [21, 22]. Currently, there are three heparan sulfate mimetics that are in early stage clinical trials in cancer patients; Roneparstat, PG545 and M402 [12, 23, 24]. Though all these inhibitors have been shown to improve chemotherapy, their use for blocking drug resistance has never been addressed. By demonstrating that heparanase is elevated in tumor cells that survive even the most intensive therapy in myeloma patients, our present study has unveiled the importance of heparanase in the outcome of anti-myeloma therapy. The inhibitor used in the current study, Roneparstat, was previously shown to have moderate anti-tumor effects as a single agent against established myeloma tumors growing subcutaneously [11]. However, the present work establishes that Roneparstat is highly effective when used in combination with a proteasome inhibitor or melphalan, even against established and aggressive tumors growing within bone (Figure 4). Thus, these findings support the use of Roneparstat in combination with other anti-myeloma drugs as a novel therapeutic strategy to inhibit disseminated tumor growth and overcome drug resistance in myeloma. Our data also point to the potential use of heparanase inhibitors like Roneparstat for targeting minimal residual disease (MRD) in myeloma patients, the idea being that inhibition of heparanase might interfere with reestablishment of a microenvironment, thereby preventing relapse. Recent studies have also demonstrated the use of Roneparstat to block chondrogenesis that drives benign cartilaginous tumors in the skeletal disorder, hereditary multiple exostoses [25]. Because heparanase promotes metastasis [26] RONEPARSTAT could also be potentially used to target heparanase-driven metastasis in different cancers [27]. In summary, our data provide new insight into heparanase mechanism of action in cancer and reveal the potential of anti-heparanase therapy to enhance response to chemotherapy and to prevent tumor relapse, thus improving patient outcome.

## MATERIALS AND METHODS

### Cell lines and reagents

MM1.S human myeloma cell line was obtained in 2007 from Drs. Nancy Krett and Steven Rosen, Northwestern University. CAG human myeloma cells were isolated in the laboratory of Dr. Joshua Epstein, University of Arkansas for Medical Sciences, Little Rock and obtained in 1999 [28]. All the cell lines were expanded and frozen in multiple vials upon receipt. All experiments were carried out within six weeks of thawing the cells. Although the authors have not authenticated these cell lines, the CAG cells continue to form tumors *in vivo* and secrete kappa immunoglobulin light chain. RPMI-8226 and U266 were obtained from the American Type Culture Collection (Manassas, VA). Generation of CAG cells expressing high (HPSE-high) and low (HPSE-low) levels of heparanase have been described earlier [10]. To express luciferase, cells were mixed with 50 μl of lentiviral particles bearing the luciferase gene (lentivirus kindly provided by Dr. John Kappes, UAB) and selected using puromycin. It was confirmed that expressing luciferase did not alter growth rate or the expression of cell associated markers (shed syndecan-1, HGF, VEGF, MMP-9, and heparanase; data not shown). Myeloma cell lines were grown in RPMI 1640 medium supplemented with 10% fetal bovine serum (FBS). Reagents utilized were as follows: bortezomib, carfilzomib, and selumetinib (SelleckChem); melphalan, dexamethasone, actin antibody (Sigma-Aldrich); PD98059, U0126 (Calbiochem), anti-ubiquitin antibody (Santa Cruz). Roneparstat is a proprietary drug of sigma-tau Research Switzerland S.A. and is currently in phase I trials in advanced multiple myeloma patients (ClinicalTrials.gov Identifier: NCT01764880).

### Studies using human subjects

Myeloma patient samples were collected at the Huntsman Cancer Institute, University of Utah according to protocol 25009. All the studies were approved by the Institutional Review Board of the University of Utah. Informed consent was obtained in accordance with the Declaration of Helsinki. Procedures for plasma cell purification, gene expression profiling (GEP) of samples and data analyses are as described in [29, 30]. Briefly, all patients received one induction cycle with D-PACE (dexamethasone, cisplatin, adriamycin, cytoxan, etoposide) followed 5–6 weeks later by treatment with velcade (bortezomib), thalidomide, dexamethasone and melphalan (VTD-MEL 200) and the first autologous transplant. Approximately 2.5 to 3 months after the first transplant patients were treated with velcade, gemcitabine, dexamethasone, BCNU and melphalan followed by a second autologous transplant. The samples were taken for GEP just before D-PACE (baseline), 5 weeks post-D-PACE (Post-induction), 2.5 months after the first transplant (Post-1st) and 2 months after the second transplant (Post-2nd).

### Animal studies

CB.17/ICR SCID male mice (5–6 weeks, ~20 g) were obtained from Charles River Breeding Laboratories and housed and monitored in the animal facility at UAB. All the animals were handled as per protocols and procedures approved by the UAB Institutional Animal Care and Use Committee. To establish disseminated myeloma tumors, 3 × 10^6^ CAG HPSE-high cells were injected into the lateral tail vein of SCID mice. Injections with different chemotherapeutic drugs or Roneparstat were initiated one week after injecting tumor cells. In all the animal experiments bortezomib and melphalan were administered intraperitoneally and Roneparstat was injected subcutaneously. The animals were monitored regularly, weighed, and imaged weekly for bioluminescence using an IVIS-100 system (Xenogen Corporation). At the end of the treatment period, sera were collected from all the animals and level of human immunoglobulin κ light chain, a measure of whole animal tumor burden was determined by ELISA (Bethyl Laboratories).

Quantification of whole body bioluminescent images from animals was performed using Living Image^®^ software. Briefly, animals were anesthetized under isoflurane after intraperitoneal injections of D-luciferin. Images were collected from individual animals from both ventral and dorsal positions for each imaging session in a Xenogen, IVIS-100 imaging system. The light emitted from the bioluminescent tumors was detected using a cooled charge-coupled device camera mounted on a light-tight specimen box. Identical regions of interest, exposure and image settings were used while analyzing animals of the same experiment to quantify total bioluminescence.

### Viability and apoptosis assays

Myeloma cells were seeded at a concentration of 3.0 × 10^4^ cells/well (MTT), 10^6^ cells/ml (apoptosis assay), 0.5 × 10^6^ cells/ml (ATP-lite assay) and treated with different concentrations of drugs for the specified length of time. Use of a particular drug concentration for an individual assay was determined based on the a) the sensitivity of cell lines to a particular drug, b) number of cells used in the assay, and c) the length of incubation. Based on the above parameters, we determined the optimal concentration of each drug for individual assays and in some cases have used different concentrations of the same drug between different types of assays. Vehicle treated cells served as the control. Cell viability was measured by CellTiter 96^®^ Non-Radioactive cytotoxicity assay, MTT (Promega) as per manufacturer's instructions. Staining for apoptosis using an Annexin V apoptosis detection kit (BD Biosciences) and testing cell viability using ATP-Lite assay (Perkin Elmer) were done according to the manufacturer's instructions.

### Western blotting

Cell lysates were prepared by incubating cell pellets with lysis buffer (50 mM Tris, pH 7.5, 150 mM NaCl, 0.5% Triton X-100) containing 1 × HALT protease and phosphatase inhibitor mixture (Pierce) for 30 min on ice. Lysates were centrifuged at 12,000 × *g* at 4°C for 15 min, supernatants were separated and their total protein concentration was determined by BCA assay (Pierce). Equal amounts of total protein were then loaded onto 4–20% gradient SDS-polyacrylamide gels (Bio-Rad), transferred to a nitrocellulose membrane (Schleicher & Schuell) and probed with specific primary antibodies followed by horseradish peroxidase-conjugated secondary anti-mouse antibody (GE Healthcare). β-actin was used as loading control. Immunoreactive bands were probed using enhanced chemiluminescence (GE Healthcare).

### Statistical analyses

All statistical analyses were done using Graph Pad Prism software. The error indicates the standard error mean (SEM). All animal experiments were conducted using at least 8 mice per group. Statistical evaluation of data was carried out using student's *t*-test and values that showed *P* < 0.05 were considered statistically significant. For values that were not normally distributed the Mann-Whitney rank sum test was used for statistical evaluation.

## SUPPLEMENTARY MATERIAL FIGURES AND TABLES


