# Supplementary material for: Targeting heparanase overcomes chemoresistance and diminishes relapse in myeloma
Source: Oncotarget. 2015 Nov 27;7(2):1598–607. doi: 10.18632/oncotarget.6408 (PMC4811483; doi:10.18632/oncotarget.6408)
Supplement: Supplementary file 1 [file oncotarget-07-1598-s001.pdf]

Targeting heparanase overcomes chemoresistance and diminishes relapse in myeloma

Supplementary Materials

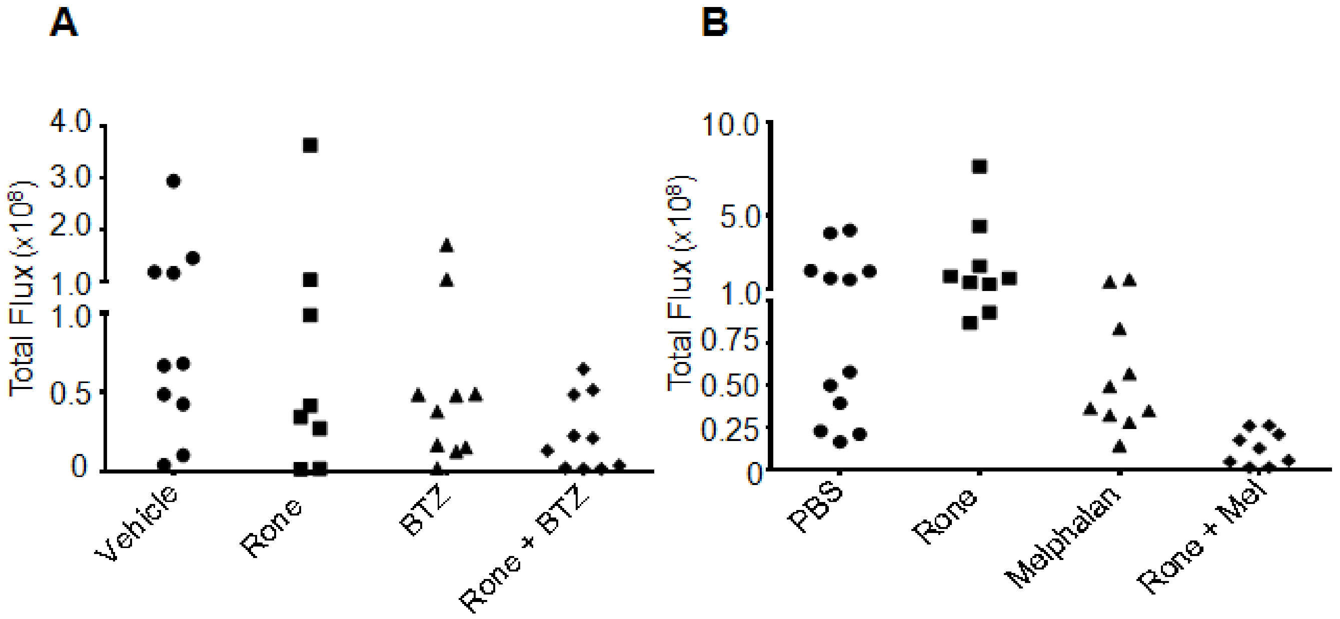

Supplementary Figure S1: Quantified data from whole body bioluminescent images of animals from combination therapy experiments shown in Figure 4C. Graphs represent whole body bioluminescence data from individual animals in different experiments presented as total flux, quantified using the Living Image® software (dorsal view, 60s exposure, 1000 min −10000 max range). Bioluminescent images used for quantitation were from day 21 for (**A**, **B**).

Supplementary Table S1: Fold increase in heparanase expression over consecutive cycles of chemotherapy

Patient ID	% Baseline		
	Post-induction	Post-1st	Post-2nd
**P01**	158.4	906.4	2524.0
**P02**	215.5	1254.6	4484.9
**P03**	342.7	770.9	738.0
**P04**	135.2	8172.7	2461.1
**P05**	489.1	1614.4	1737.6
**P06**	249.6	823.7	481.5
**P07**	350.8	2827.8	78.2
**P08**	158.2	146.3	322.2
**P09**	138.2	87.1	89.1

In the patients tested, fold increase in heparanase expression after different rounds of chemotherapy was calculated by normalizing to their corresponding baseline heparanase expression (heparanase levels at baseline was set to 100% for individual patients). Results revealed that compared to baseline, heparanase expression was elevated in all the patients after the induction therapy, in eight out of nine patients after the first round of high dose chemotherapy, and in in seven out of nine patients after the second round of therapy.
